# *BRAF* alteration status and the histone *H3F3A* gene K27M mutation segregate spinal cord astrocytoma histology

**DOI:** 10.1007/s00401-015-1492-2

**Published:** 2015-10-20

**Authors:** Ganesh M. Shankar, Nina Lelic, Corey M. Gill, Aaron R. Thorner, Paul Van Hummelen, Jeffrey H. Wisoff, Jay S. Loeffler, Priscilla K. Brastianos, John H. Shin, Lawrence F. Borges, William E. Butler, David Zagzag, Rachel I. Brody, Ann-Christine Duhaime, Michael D. Taylor, Cynthia E. Hawkins, David N. Louis, Daniel P. Cahill, William T. Curry, Matthew Meyerson

**Affiliations:** Cancer Program, Broad Institute, Cambridge, MA USA; Department of Neurosurgery, Massachusetts General Hospital, 32 Fruit Street, YAW-9-9040, Boston, MA 02114 USA; Division of Neuro-Oncology, Massachusetts General Hospital, Boston, MA USA; Center for Cancer Genome Discovery, Dana Farber Cancer Institute, Boston, MA USA; Medical Oncology, Dana Farber Cancer Institute, Boston, MA USA; Department of Neurosurgery, New York University Langone Medical Center, New York City, NY USA; Department of Radiation Oncology, Massachusetts General Hospital, Boston, MA USA; Department of Pediatric Laboratory Medicine, University of Toronto, Toronto, ON Canada; Department of Pathology, Massachusetts General Hospital, Boston, MA USA; Department of Pathology, New York University Langone Medical Center, New York City, NY USA; Division of Neurosurgery, Arthur and Sonia Labatt Brain Tumour Research Centre, The Hospital for Sick Children, Toronto, ON Canada; Dana Farber Cancer Institute, Harvard Medical School, 450 Brookline Avenue, Boston, MA 02215 USA

Intramedullary spinal cord neoplasms represent 2–4 % of central nervous system tumors, of which astrocytic gliomas represent 80 %. Patients presenting with spinal cord astrocytomas span the traditional pediatric and adult age divisions, having an overall age-distribution that is younger than cohorts with supratentorial gliomas. WHO grade I and II astrocytomas have better outcomes that are largely dependent on extent of surgical resection [10], whereas Grade III and IV astrocytomas are less amenable to safe surgical resection, and typically require adjuvant radiation and chemotherapy for treatment. Given the premium on preserving neurologic function during spinal cord surgery, intraoperative frozen section histologic analysis has an important role in driving therapeutic decision-making. However, histologic grading can be challenging in spinal cord astrocytomas because of the often relatively small samples obtained at the time of the surgical procedure. Therefore, grade-defining molecular biomarkers would be particularly useful for the accurate diagnostic classification of these tumors [13]. Recent genome level sequencing studies of supratentorial gliomas revealed discrete genomic alterations that discriminate pilocytic astrocytomas, WHO grade II and III diffuse gliomas, and WHO grade IV glioblastoma (GBM), with notable differences between pediatric [9, 14, 15, 20] and adult [2, 3, 6] patients. To address the hypothesis that genomic alterations could segregate spinal cord astrocytoma histologic grades, we performed sequencing of cancer-related genes in a cohort of 17 tumors.

Spinal cord astrocytomas from children and adults were obtained as formalin-fixed, paraffin-embedded (FFPE) specimens from Massachusetts General Hospital, the University of Toronto, and New York University. Central neuropathology review performed by a neuropathologist (DNL) and specimens with clear histologic diagnosis and grading were used for further analysis. The characteristics of the discovery cohort (*n* = 17 specimens) are listed in Table 1. Targeted sequencing of 560 cancer related genes and 39 translocation events was performed on DNA extracted from these specimens (Supplementary Table 1) [5]. Briefly, DNA was sonicated to achieve an average fragment size of 250 base pairs, size selected and barcoded. Multiplexed pools were hybridized with biotinylated baits (Agilent SureSelect) designed to capture exonic sequences. The captures were sequenced on the Illumina HiSeq 2500 in Rapid Run Mode. Mutation analysis was performed by MuTect [4] and SomaticIndelDetector, copy number variant analysis was performed by ReCapSeg, and rearrangement analysis was performed by BreaKmer [1]. When applicable, statistical comparisons were performed by Chi squared test.

**Table 1:** Baseline characteristics of discovery cohort

Specimen	Age (years)	WHO grade	Gender
SA-TL04	5.2	I	Female
SA-TL13	4.5	I	Male
SA-TL11	13.6	I	Female
SA-TL19	5.4	I	Female
SA-TL20	5.8	I	Male
SA-TL14	5.9	I	Male
SA-TH04	17.2	I	Female
SA-TL02	8.3	I	Male
SA-TL12	9.0	I	Male
SA-TL17	13.4	I	Female
SA-TL10	1.5	II	Male
SA-N101	82.0	II	Female
SA-TL07	2.2	II	Female
SA-TL03	14.6	III	Female
SA-N103	25.0	IV	Male
SA-TH01	2.9	IV	Male
SA-TH02	12.3	IV	Male

The most recurrent findings in Grade I spinal cord astrocytomas were a *BRAF*-*KIAA1549* translocation (*n* = 3/10) and *BRAF* copy number gain (*n* = 5/10) (Fig. 1). Additionally, WHO grade I astrocytomas were found to have non-synonymous mutations in *NF2*, *NTRK1*, *NTRK3*, *PDGFRA*, and *TP53* (Supplementary Table 2). WHO grade II astrocytomas were similarly characterized by alterations involved in the MAPK-ERK or PI3K pathways, including *BRAF*-*KIAA1549* translocation (*n* = 1/3) and *BRAF* amplification (*n* = 2/3). For samples with sufficient material, low coverage whole genome sequencing (mean 1× depth) was performed revealing that the *BRAF* amplification resulted from a chromosome 7 arm level gain in three of these specimens (SA-N101, SA-TL07, and SA-TL17, Supplementary Figure 1). Notably, no specimen in the discovery cohort was characterized by the *BRAF* V600E mutation.

**Fig. 1:**
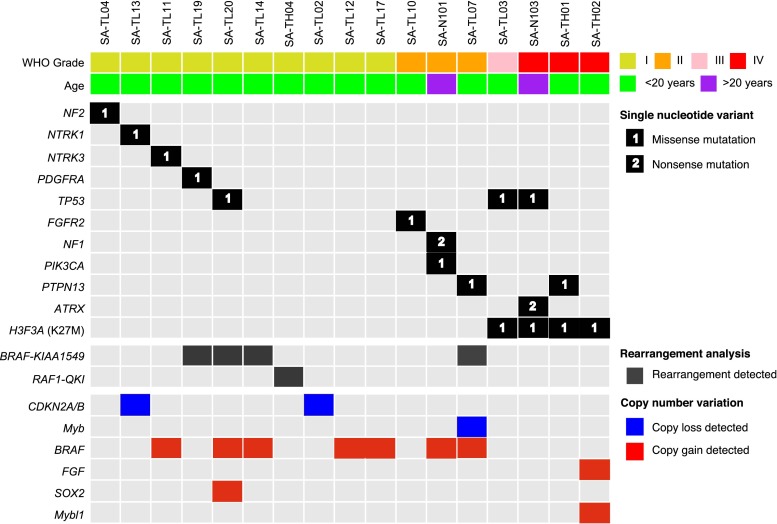
Exomic characterization of spinal cord astrocytomas reveals that *BRAF* alterations and the *H3F3A* K27M mutation segregate histologic grade. Central neuropathology review was performed on the cohort of specimens used in this study (*top row*). Grade I and II astrocytomas were notable for genome alterations in genes involved in the *MAPK*-*ERK* and *PI3K* pathway, whereas *H3F3A* K27M mutation was detected exclusively in Grade III and IV astrocytomas. *BRAF*-*KIAA1549* was observed in 4/10 Grade I and II astrocytomas. Copy number analysis revealed amplification of *BRAF* in 7/13 Grade I and II specimens

In addition, we observed that all four Grade III and IV astrocytomas in the discovery cohort shared the *H3F3A* K27M mutation. Further targeted Sanger sequencing of *H3F3A* was performed in five additional specimens (validation cohort) and revealed the K27M mutation in 2/3 spinal Grade IV astrocytomas and 0/2 Grade I astrocytomas (Supplementary Figure 2). The age distribution of our findings are consistent with prior observations that *H3F3A* K27M primarily occurs in pediatric and young adult gliomas [11, 15, 19]. In the aggregate cohort of 22 specimens (discovery and validation cohorts), the presence of *H3F3A* K27M in Grade III and IV (85.7 %, *n* = 6/7 specimens) and absence in Grade I and II (*n* = 0/15 specimens) astrocytomas was a statistically significant difference (*p* < 0.001, Chi squared test with Yates correction).

Of note, while variants in *IDH1* and *IDH2* were noted in four specimens (Supplementary Table 2), none of these represented the recurrent mutations previously described in adult glioma. Loss of heterozygosity analysis of variant allele frequency [17] did not reveal co-deletion of chromosomes 1p and 19q (Supplementary Figure 3), further confirming that the tumors analyzed in the discovery cohort were astrocytic.

The distribution of mutations observed may partially underlie the well-established demographic differences between patients with spinal cord gliomas compared to their supratentorial counterparts. For instance, whole genome analyses of pediatric intracranial gliomas have been reported recently with convergence of alterations in Grade I and II gliomas on MAPK-ERK and PI3K pathways [9]. Pediatric high grade gliomas, on the other hand, have been characterized by recurrent mutations in chromatin remodeling genes *H3F3A*, *ATRX*, and *DAXX* in 44 % of sequenced tumors [15]. Similarly, seminal work revealed that *H3F3A* K27M is found in 71 % of pediatric diffuse intrinsic pontine glioma, the presence of which correlated with worse outcomes [11]. Across pediatric and young adult GBM, *H3F3A* K27M mutations occur mutually exclusive of other category-defining recurrent mutations (such as mutations in *IDH1* and *TERT* promoter) and are found predominantly in midline lesions bearing the transcriptomic profile of the proneural GBM subtype [18]. A recent report noted positive *H3F3A* K27M immunohistochemical staining in 11 spinal glioblastomas, 3 anaplastic astrocytomas, and 2 anaplastic gangliogliomas [7]. Together with our observation of *H3F3A* K27M occurring in 86 % of Grade III and IV spinal cord astrocytomas, this supports the concept of a shared teleology between aggressive astrocytic gliomas arising in midline structures of the craniospinal axis. Future transcriptional analysis of spinal cord astrocytomas can assess whether these lesions share similar changes noted in *H3F3A* K27M mutant supratentorial gliomas.

The *BRAF* alterations in a high percentage of WHO grade I and II spinal cord astrocytomas point towards a potential therapeutic approach, as BRAF–MEK inhibitors have demonstrated success in *BRAF*-mutant cancer types. Accordingly, targeting the BRAF–MEK pathway in pediatric gliomas is under active evaluation [12]. Our findings suggest that patients with spinal cord astrocytomas could be considered for enrollment in clinical trials targeting these pathways. From a surgical management standpoint, the hotspot *H3F3A* K27M mutation has the potential to be genotyped within an intraoperative timeframe, to guide the aggressiveness of surgical resection by balancing the neuromonitoring-based potential for postoperative neurologic deficit with the predicted natural history defined by *H3F3A* K27M mutation status [16]. Detection of this mutation could ultimately guide novel adjuvant treatment strategies, as inhibition of histone deacetylase and histone demethylase has demonstrated in vivo efficacy in xenografts of H3F3A K27M mutant gliomas [8].

While our findings do not indicate alterations specific to spinal cord astrocytomas versus supratentorial disease, larger cohort studies performing deep coverage whole genome or transcriptome may reveal unique copy number alterations or translocations in these infiltrative tumors. In summary, the findings described here indicate that *BRAF* alterations and histone *H3F3A* K27M mutations are grade-related features of spinal cord astrocytomas that should enter routine initial evaluation of spinal cord gliomas, and provide a potential foundation for adjuvant therapeutic strategies.

## Electronic supplementary material

Supplementary material 1 (DOCX 11 kb)

Supplementary material 2 (XLSX 65 kb)

Supplementary material 3 (PDF 384 kb)
