# Supplementary material for: BRAF alteration status and the histone H3F3A gene K27M mutation segregate spinal cord astrocytoma histology
Source: Acta Neuropathol. 2015 Oct 20;131:147–50. doi: 10.1007/s00401-015-1492-2 (PMC4698284; doi:10.1007/s00401-015-1492-2)
Supplement: Supplementary file 1 — Supplementary material 1 (DOCX 11 kb) [file 401_2015_1492_MOESM1_ESM.docx]

Supplementary Figure 1. Low coverage whole genome sequencing reveals that *BRAF* amplification may result from arm level changes in chromosome 7. Specimens with sufficient material were subjected to low coverage whole genome sequencing (WGS) at mean 1x depth. Copy number analysis in tumor specimens was performed by Nexus 7.1. Analysis parameters were as follows: Sequential Loess correction with 0.2 smoothing window; significance threshold = 5E-6; max contiguous probe spacing of 1000 Kbp; minimum number of probes per segment = 3. Gains were called for log ratio >0.3 and were called high gain if >0.6. WGS log ratio plot for sample SA-N101 with noted gain of chromosome 7 is demonstrated in this figure.

Supplementary Figure 2. Targeted Sanger sequencing of spinal cord astrocytomas reveals *H3F3A* K27M mutation is specific to pediatric spinal cord GBM.

Supplementary Figure 3. Spinal cord gliomas analyzed in this study have retained chromosome 1p and 19q. The frequency of allele fractions of mutations on chromosome 1p and 19q were plotted and loss of heterozygosity (LOH) of 19q was noted in only one sample (SA-N103) where allelic shift was noted from the expected distribution centered at 0.5. No samples were notable for co-deletion of chromosomes 1p and 19q. Inset legend refers to the number of sequenced alleles available for the LOH distribution analysis.

Supplementary Table 1. List of exomes and translocations that were sequenced in the discovery cohort. Coding regions of 560 genes and 39 translocations previously implicated in cancer were targeted in the analysis of this cohort.

Supplementary Table 2. Composite table of the somatic mutations detected in the discovery cohort by targeted sequencing of 560 cancer associated genes.
