# Supplementary figures and images for: BRAF alteration status and the histone H3F3A gene K27M mutation segregate spinal cord astrocytoma histology
Source: Acta Neuropathol. 2015 Oct 20;131:147–50. doi: 10.1007/s00401-015-1492-2 (PMC4698284; doi:10.1007/s00401-015-1492-2)

Supplementary Figure 1

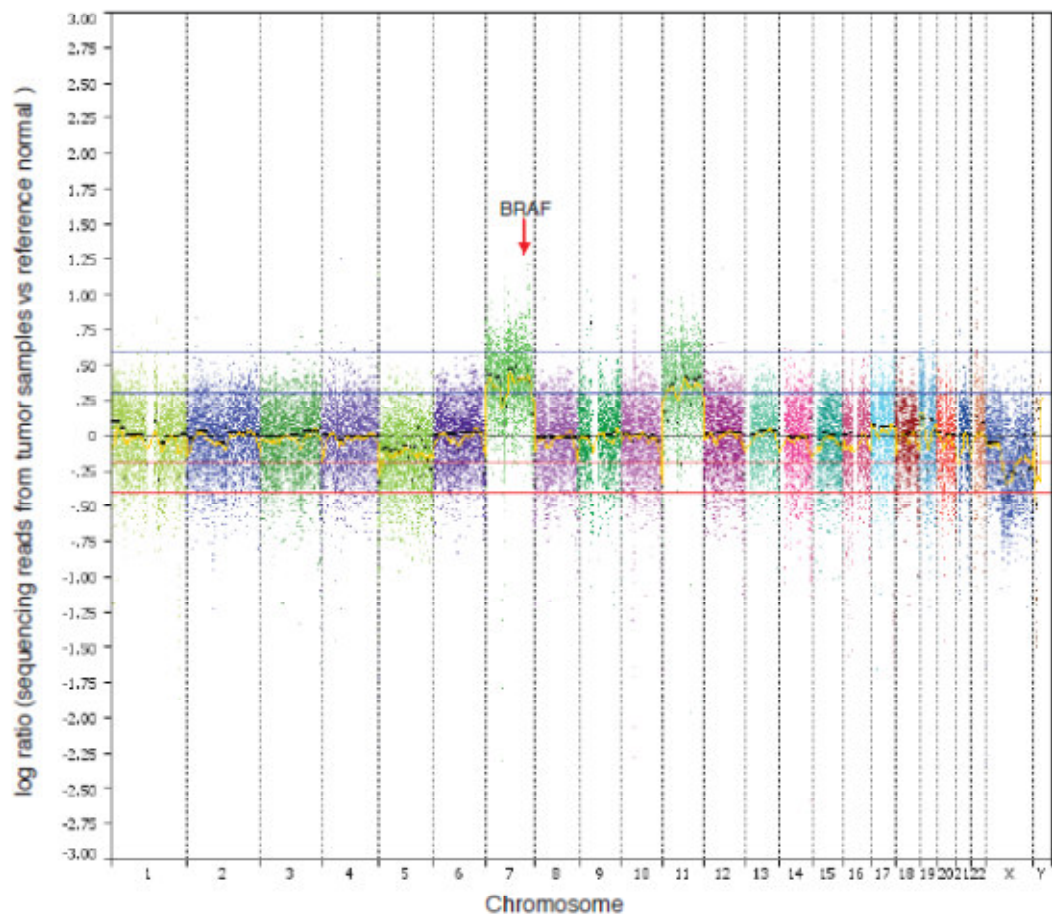

Supplementary Figure 2

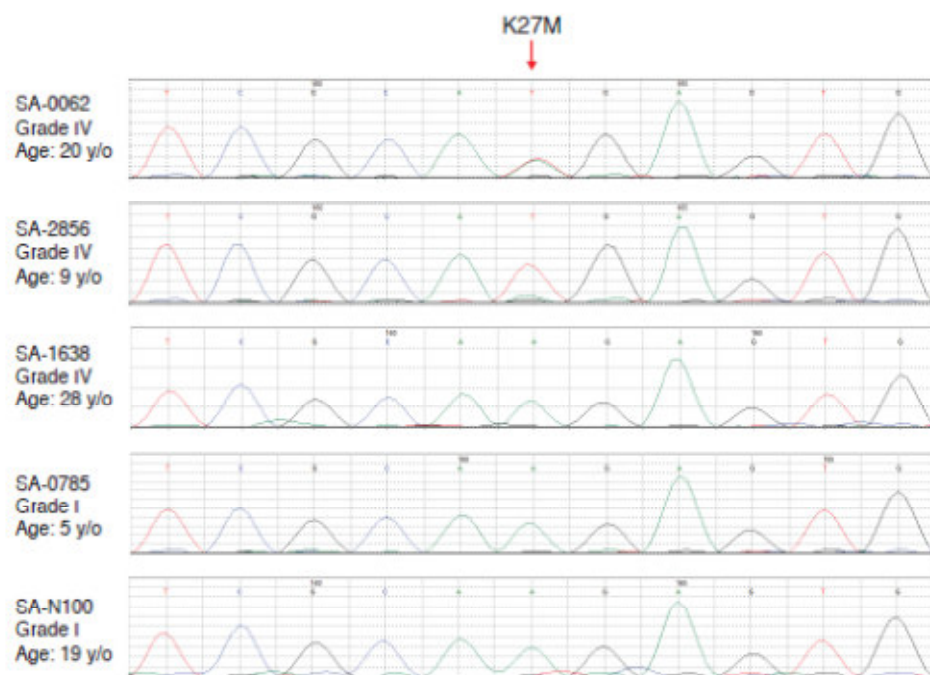

Supplementary Figure 3

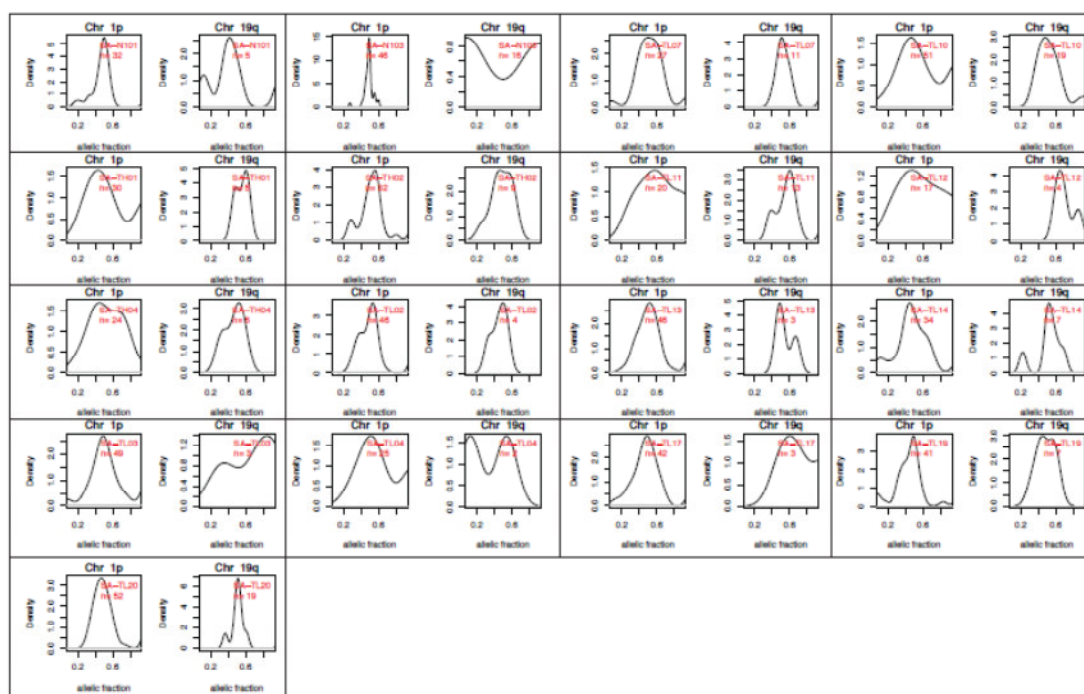

Supplement: Supplementary file 3 — Supplementary material 3 (PDF 384 kb) [file 401_2015_1492_MOESM3_ESM.pdf]
